# Glances at the Hospitals

**Published:** 1904-05-21

**Authors:** 


### GLANCES AT THE HOSPITALS.

### CUMBERLAND INFIRMARY.

THE ordinary income amounted to £4,980, and this included a donation of £1,000 from Mrs. James Carr. The ordinary expenditure was £6,079. Taking the total expenditure and the total income it is found that there was a balance of nearly £500 on the wrong side, and the managers say that the financial position is such that it must have the serious consideration of the committee and the public. There was a total indebtedness at the end of the year of £1,870, but most of this was incurred during the previous year, or at least was standing over from that year. The demand for beds was on many occasions in excess of the accommodation; and we know how painful it would be to the committee of any hospital to refuse admission to patients; and while it is the duty of the public to come forward with subscriptions it is equally the duty of the managers to keep the expenditure within their income. The average cost per bed occupied was £63 8s., and it cannot be said that this is excessive, compared with other hospitals, for although some, such as Derby and Hull, are lower, a good many are higher. On January 1st the hospital contained 83 patients, and 866 were admitted during the year, making a total of 949 under treatment. Of these 760 were discharged cured or relieved, 33 were discharged for other reasons, 75 died and 81 remained in the wards at the end of the year. The average stay in the hospital was rather high, namely, 35 days, nearly. Excepting that there is no table of the anæsthetics used, the medical and surgical statistics are satisfactorily given. There are separate columns for the "cured," "relieved," "incurable," and "died," so that it is easy to arrive at the results of treatment. For instance, there were 26 cases of abdominal section. Of these 17 recovered, 7 died, and 2 remained under treatment.

### WESTERN INFIRMARY, GLASGOW.

The ordinary income amounted to £20,716, and the ordinary expenditure to £30,269, showing a deficit of £9,553. The extraordinary income was £7,926, and the extraordinary expenditure was £4,192. After using the balance on this account, there was still a deficit of £6,545, which had to be met from the stock account, and this has absorbed more than one-fifth of the available capital funds. The managers say that if the efficiency and usefulness of the institution are to be maintained, there must be a large increase in the subscriptions, and it is clear enough that the capital account cannot be annually drawn on to any great extent. The managers also look to their scheme of endowment of beds to relieve them of their embarrassments; and two beds have been so endowed during the past year, one by Miss Gilchrist and one by Sir James Thompson, but two or three beds a year will not do much for them for a while at least, and in the meantime either subscriptions must flow in or wards must be closed, for surely no board of managers will sanction such a rapid consumption of capital as had to be carried out lately. At the beginning of the year the hospital contained 444 patients, and 5,484 were admitted during the year. Of the total, 2,941 were discharged cured, 176 greatly improved, 1,169 improved, 715 on other grounds, 488 died, and 439 remained under treatment. The statistics are clearly arranged. There were 135 cases of abdominal section, and of these 98 recovered and 37 died. Of 70 cases of acute rheumatism, 68 were cured and 2 died. We notice that 18 cases of aortic aneurism were treated during the year, and all these are put down in column for "cured or relieved," and we think that in these cases an explanatory marginal note would have been desirable. There were 149 cases of pneumonia, resulting in 114 recoveries and 35 deaths.

### THE QUEEN'S HOSPITAL, BIRMINGHAM.

The income was £10,283, and the expenditure £11,066. The average daily number of patients was 123, the cost per patient was £3 12s., the average stay in the hospital was nearly 23 days, and the "death-rate per bed" was 1·7. The death-rate calculated on the number of patients was 11 per cent. The total number of beds is 132, and the total number of in-patients was 2,065. Of these 1,335 were discharged recovered, 341 relieved, 65 not relieved, 216 died, and 108 remained under treatment. There is no anæsthetic table, but otherwise the statistics convey all necessary information. Of 27 cases of pneumonia 14 were cured, 3 relieved, 8 died, and 2 remained under treatment. Of 65 cases of appendicitis 53 were cured, 1 was relieved, 10 died, and 1 remained in the hospital. The operations are detailed under the names of the surgeons under whose care the patients were placed. The operations in the eye department and in the department for diseases of women are given separately.

